# Secreted Frizzled-Related Protein 2 (sFRP2) promotes osteosarcoma invasion and metastatic potential

**DOI:** 10.1186/s12885-016-2909-6

**Published:** 2016-11-08

**Authors:** Piti Techavichit, Yang Gao, Lyazat Kurenbekova, Ryan Shuck, Lawrence A. Donehower, Jason T. Yustein

**Affiliations:** 1 Texas Children’s Cancer and Hematology Centers, Department of Pediatrics, Baylor College of Medicine, Houston, TX 77030 USA; 2 Department of Pediatrics, Hematology-Oncology, Bumrungrad Hospital, Bangkok, Thailand; 3 Integrative Molecular and Biological Sciences Program, Baylor College of Medicine, Houston, TX 77030 USA; 4 Department of Molecular and Cellular Biology, Baylor College of Medicine, Houston, TX 77030 USA; 5 Department of Molecular Virology & Microbiology, Baylor College of Medicine, Houston, TX 77030 USA

## Abstract

**Background:**

Osteosarcoma (OS), which has a high potential for developing metastatic disease, is the most frequent malignant bone tumor in children and adolescents. Molecular analysis of a metastatic genetically engineered mouse model of osteosarcoma identified enhanced expression of Secreted Frizzled-Related Protein 2 (sFRP2), a putative regulator of Wnt signaling within metastatic tumors. Subsequent analysis correlated increased expression in the human disease, and within highly metastatic OS cells. However, the role of sFRP2 in osteosarcoma development and progression has not been well elucidated.

**Methods:**

Studies using stable gain or loss-of-function alterations of sFRP2 within human and mouse OS cells were performed to assess changes in cell proliferation, migration, and invasive ability in vitro*,* via both transwell and 3D matrigel assays. In additional, xenograft studies using overexpression of sFRP2 were used to assess effects on in vivo metastatic potential*.*

**Results:**

Functional studies revealed stable overexpression of sFRP2 within localized human and mouse OS cells significantly increased cell migration and invasive ability in vitro and enhanced metastatic potential in vivo. Additional studies exploiting knockdown of sFRP2 within metastatic human and mouse OS cells demonstrated decreased cell migration and invasion ability in vitro*,* thus corroborating a critical biological phenotype carried out by sFRP2. Interestingly, alterations in sFRP2 expression did not alter OS proliferation rates or primary tumor development.

**Conclusions:**

While future studies further investigating the molecular mechanisms contributing towards this sFRP2-dependent phenotype are needed, our studies clearly provide evidence that aberrant expression of sFRP2 can contribute to the invasive and metastatic potential for osteosarcoma.

**Electronic supplementary material:**

The online version of this article (doi:10.1186/s12885-016-2909-6) contains supplementary material, which is available to authorized users.

## Background

Osteosarcoma is the most common frequent malignant bone tumor within the pediatric population [1, 2]. Metastasis to the lungs or other bones is a poor prognostic indicator with long term overall survival rate of 10–30 % [3, 4]. Understanding disease biology and the molecular signaling pathways involved in osteosarcoma development and progression should lead to the identification of novel therapeutic targets.

The Wingless (Wnt) signaling pathway is involved in normal embryonic development [5, 6]. Wnt activity is regulated at the cell membrane by a complex network of transmembrane proteins [7, 8]. For the canonical signaling pathway, binding of Wnt ligands to Frizzled receptors, which are G protein-coupled receptors, leads to activation and translocation of β-catenin from the cytoplasm to nucleus. Subsequent binding to T cell factor (TCF)/lymphoid enhancer factor (LEF) leads to transcriptional activation of downstream target genes [9, 10]. However, aberrant activation of Wnt signaling pathways has been reported in many types of cancer including colorectal, brain, and sarcomas [7, 11].

The Wnt signaling pathway is partially regulated by extracellular Wnt antagonists, consisting of members of Dickkopf and secreted Frizzled-related proteins (sFRPs) families, and Wnt inhibitory factor 1 [12, 13] The sFRP family consists of five different glycoproteins (sFRP1-5), each containing a highly homologous cysteine-rich domain and putative binding site on the Frizzled receptor binding site used by the Wnt ligands. Consequently, the role of sFRPs has been predominantly focused on preventing Wnt ligands from binding the Frizzled receptors, which results in Wnt signaling downregulation [9, 14].

Specifically, using insights gained from our genetically engineered mouse model (GEMM) of metastatic OS and correlative human studies, we have identified aberrant expression of sFRP2 in metastatic osteosarcoma [15]. The dysregulation of sFRP2 has been reported in several malignancies, however the mechanisms by which it contributes to the biology of these cancers has been variable. For instance, sFRP2 is noted to be downregulated via epigenetic hypermethylation in human gastric cancer [16], colorectal cancer [17, 18], and oral squamous cell carcinoma [19, 20] suggesting a role as a tumor suppressor. However, overexpression of sFRP2 has been reported in renal cancer [8], human angiosarcoma, and breast cancer [21, 22], which leads to angiogenesis stimulation by activation of the calcineurin/NFATc3 pathway. Furthermore, recently enhanced sFRP2 expression has been associated with promoting therapeutic resistance and metastatic potential within solid tumors by specifically altering the tumor microenvironment [23, 24].

To our knowledge, the functional significance of sFRP2 in osteosarcoma has not been well studied. Our studies provide insights into the functional role of sFRP2 within osteosarcoma tumor development and metastasis. We demonstrate that sFRP2 expression strongly enhances metastatic potential both in vitro and in vivo, but has no noted effects on tumor cell proliferation or primary tumor development. Further studies are warranted to investigate the exact mechanisms of action for sFRP2 and its regulation of metastatic pathways for osteosarcoma.

## Methods

### Cell culture and transfections

Highly metastatic mouse OS cell lines (RF379L, and RF1044) were derived from either p53+/R172H or p53 null OS mouse models primary OS tumors and/or lung lesions using our previously established, highly metastatic Col2.3-Cre transgenic mice with osteoblast-specific Cre expression. Low metastatic mouse OS cell line (RF43) was isolated from singly floxed p53+/F-Col2.3-Cre mice as previously reported [15]. All cell lines used for functional assays were extensively characterized for their migratory, invasive, and metastatic potentials both in vitro and in vivo prior to genetic alteration, overexpression or knockdown, of SFRP2 status.

The human osteosarcoma cell lines, HOS and 143B were purchased from ATCC (Manassas, VA, USA). All human cell lines used in these studies were authenticated through STR analysis at MD Anderson (https://www.mdanderson.org/research/research-resources/core-facilities/characterized-cell-line-core-facility.html) and were tested and remained free of mycoplasma. All cells were cultured in Dulbecco’s Modified Eagle’s Medium (DMEM) supplemented with 10 % Fetal Bovine Serum (FBS) and 1 % penicillin/streptomycin (GIBCO, Life Technology, Grand Island, NY USA). All cells were allowed to grow in a humidified 37 °C, 5 % CO_2_ incubator. Stable control or sFRP2 knockdown cell lines were generated by transfecting Open Biosystems/GE Dhramacon pLKO.1 TRC control or pLKO.1 shsFRP2 vector (Clone ID: TRCN0000034474; Clone ID: TRCN0000034476) utilizing the Lipofectamine LTX reagent (Invitrogen). Positively transfected clones were selected with puromycin (2ug/mL), and real-time PCR confirmed *sFRP2* knockdown. FlexiTube (QIAGEN, catalogue# GS12393) siRNA for sFRP2 was transfected per manufacturer’s recommendation. Stable mouse and human cell lines overexpressing sFRP2 were generated by transfecting a sFRP2 or empty vector (pcDNA3.1) using the Lipofectamine LTX reagent, and positively transfected clones were selected using neomycin (1 mg/mL).

### RNA extraction and reverse transcription-PCR

Total RNA was extracted using the RNAeasy Mini Kit (Qiagen) according to manufacturer’s instructions. cDNA templates were generated by 1 μg of total RNA via RT—PCR using cDNA Synthesis Supermix (Quanta, Gaithersburg, MD, USA). The cDNA products were used for real-time PCR templates. Gene expression was monitored using real-time primer pairs with SYBR Green detection (Applied Biosystems, Life Technology) and analyzed using StepOnePlus real-time PCR machine (AB Biosystems, Life Technology). PCR conditions were as follows: 10 min at 95 °C and 60 s at 60 °C. Relative fold change in mRNA expression compared with control was calculated using ΔΔCT method. All samples were normalized to actin expression level.

### Cell proliferation assay

To assess cell proliferation, 1 × 10^3^ cells/well were seeded in a 96 well plate in 100 μl cell culture medium. Cells were cultured in a 5 % CO_2_ incubator at 37 °C. At indicated time points, viable cells were counted using Cell Counting Kit-8 (Dojindo Laboratories, Kumamoto, Japan) following the manufacturer’s protocol. The relative proliferation rates were determined by measuring the absorbance at 450 nm using a Perkin Elmer Victor2 microplate reader. Individual time points were run in triplicates. Each experiment was performed at least twice, with representative results shown.

### Cell migration assay

Cell lines, both control and sFRP2 altered, were cultured to confluence in six-well plates. Using a sterile 200-μl pipette tip, a scratch was drawn on the monolayer of the cells. The cells were then washed once with phosphate-buffered saline (PBS). Time points for analysis are indicated within figures. Assay was performed three independent times with representative images shown.

### In vitro invasion assay

The in vitro invasion assay was performed using 24-well, 8 μm pore-sized invasion chamber system (Greiner BioOne, Monroe, NC, USA) and 0.5 μg/ml collagen (Advanced Biomatrix, Carlsbad, CA, USA) monolayer. Prior to loading the cells onto the upper chamber, the lower chamber was filled with medium containing 10 % FBS. Cells were starved overnight and added to serum free medium in the upper chamber at the concentration of 1 × 10^5^ cells/well followed by incubation for 12–18 h in a humidified 5 % CO_2_ atm at 37 °C. After incubation, the cells on the upper surface of the well were removed completely by Q-tip. The wells were fixed in methanol and strained with 15 % crystal violet (Alfa Aesar, Ward Hill, MA, USA). The number of cells migrating to the lower surface of the filters were counted in five fields under 20X magnification and the mean of the number of cells in each well was calculated. Assays were performed three independent times with representative images shown.

### 3D cell morphology assay

Cells were trypsinized, placed in complete media, counted and then 10^5^ osteosarcoma cells were mixed with 3 % Matrigel in complete medium. Suspended cells were loaded into 12-well plates, allowed to polymerize at 37 °C, and then overlaid with complete media. After 72 h, phase-contrast images were taken. Assays were performed three independent times with representative images shown.

### In vivo studies

Mouse studies were approved by Baylor Animal Protocol Committee (Baylor College of Medicine Animal Protocol AN-5225. Suspensions of the stable sFRP2-expressing cell or the control cell RF43, a low metastatic cell line derived from our previously reported GEMM [15], were injected subcutaneously into female nu/nu mice (*n* = 6/cohort). Tumor size was measured with calipers once per week for six weeks and the tumor was weighed at the time of mouse sacrifice. All animals had comprehensive necropsies upon time of euthanasia with complete dissection of tumor as well as evaluation of all major organs, with special attention given to the lungs, liver and other bones. Portions of all tumor specimens and that of the lungs and liver were sent for histological analysis and evaluation of distal organs for evidence of metastatic disease. The lungs were the main site of metastasis, and lung metastatic nodules were visualized on 5 μm paraffin-embedded sections with H&E staining and were quantified.

### Western blotting

Cells were lysed in RIPA buffer (50 mM Tris-Cl, pH 7.4, 150 mM NaCl, 1 % NP-40, 0.25 % Na-deoxycholate) supplemented with protease inhibitors (Complete mini protease cocktail, Roche, Indianapolis, IN, USA) and phosphatase inhibitors (Sigma, P0044 and P5726). Nuclear lysates were isolated utilizing the NE-PER nuclear and cytoplasmic extraction reagents (Promega) following the manufacturer’s instructions. Lysates were boiled in NuPAGE LDS sample buffer (Invitrogen), separated on NuPAGE Novex 4–12 % Bis-Tris Gel (Invitrogen), and transferred to polyvinylidene difluoride membranes. The blots were probed with antibodies to sFRP2 (Company), tubulin (Fisher Scientific, Waltham, MA, USA, Ab-2, clone DM1A), or actin (EMD Millipore, Billerica, MA, USA, MAB1501, clone C4). Blots were incubated with the appropriate secondary HRP-conjugated antibodies for 1 h, and signal was detected utilizing Millipore Immobilon Western chemiluminescent HRP substrate (Millipore).

### Statistical analysis

The significance of differences between control and sFRP2 samples under different conditions was determined by Student’s *t*-test. *P* values less than 0.05 were considered statistically significant. Data are shown as mean value +/− SD.

## Results

### Identification of increased sFRP2 expression in metastatic osteosarcoma

We have previously developed and characterized a genetically engineered mouse model of metastatic osteosarcoma [15] that revealed differential expression of several categorical genes within the primary metastatic tumors, including those involved in translational regulation, inflammation, and regulation of Wnt signaling. Specifically, besides the downregulation of the negative regulators Naked2 (Nkd2) and Apcdd1, we also noted enhanced, approximately three-fold expression of sFRP2 [15]. sFRP2 is a member of a family of extracellular signaling molecules involved in the regulation of the Wnt pathway. Besides our findings, recent literature has also reported enhanced expression of sFRP2 in angiosarcoma and breast cancer, and analysis of multiple publicly available datasets (cBioPortal for Cancer Genomics, http://www.cbioportal.org) revealed relatively elevated levels of sFRP2 in sarcomas (Additional file 1: Figure S1a; red box highlighting sarcomas).

**Fig. 1:**
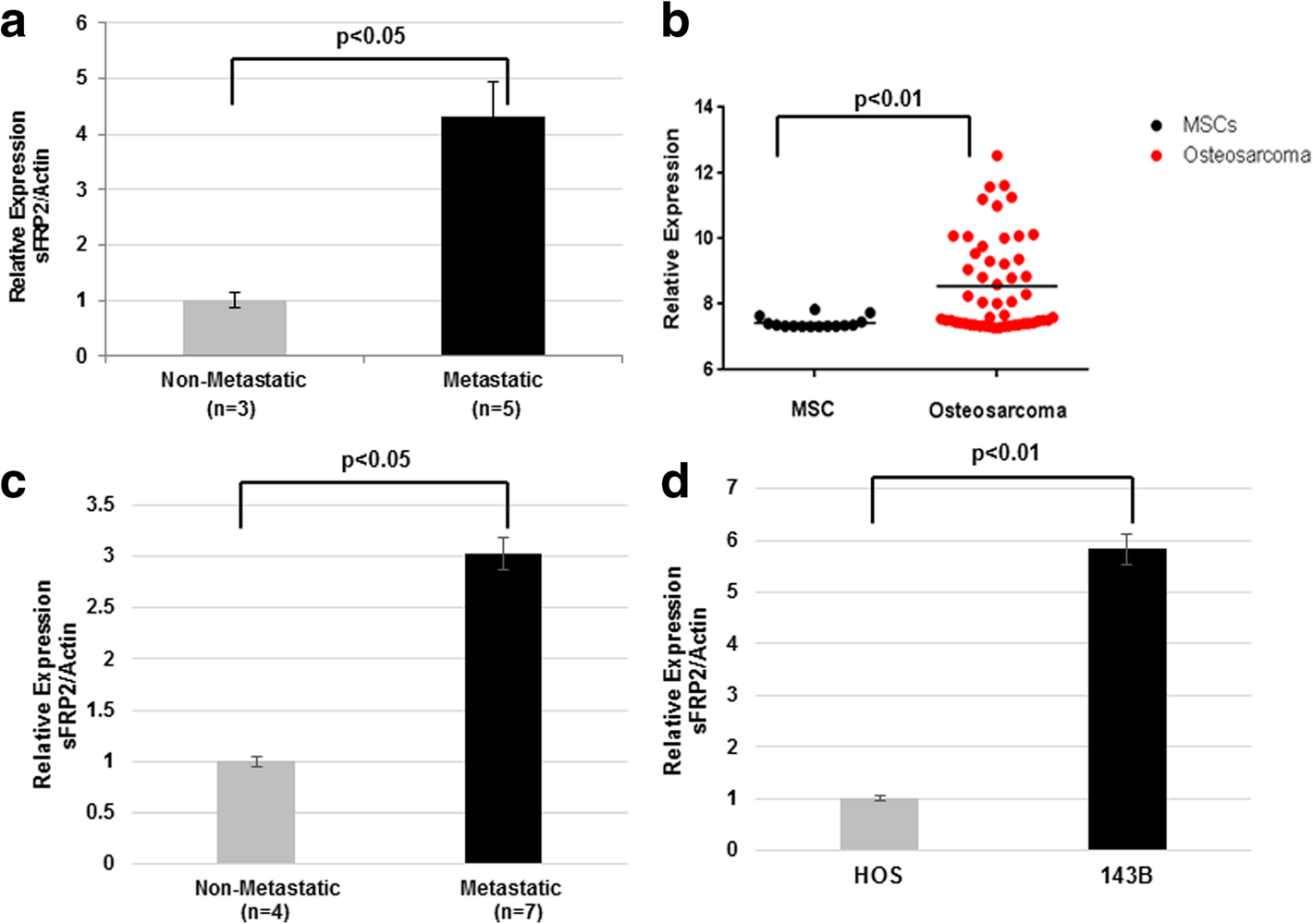
Enhanced expression of sFRP2 in metastatic osteosarcoma. **a** qPCR analysis of sFRP2 expression in mouse non-metastatic and metastatic osteosarcoma cell lines. **b** Analysis of GEO database GSE42352 for sFRP2 expression in human osteosarcoma tumors compared to mesenchymal stem cells. **c** qPCR analysis comparing expression of sFRP2 in metastatic primary human osteosarcoma tumor tissue to non-metastatic tumor. **d** qPCR analysis for human sFRP2 comparing low metastatic (HOS) versus high metastatic (143B) paired OS cell lines

To validate our initial expression profiling data, we performed qPCR of independent local and primary metastatic mouse tumors and noted significant enhanced expression of sFRP2 in the metastatic tumors (Fig. 1a). Exploration of sFRP2 expression in human osteosarcoma revealed enhanced expression compared to mesenchymal stem cells (MSCs) (Fig. 1b) [25]. We further examined the relative expression of sFRP2 in human osteosarcoma tumor specimens and cell lines. We analyzed human osteosarcoma specimens of 7 metastatic (the primary bone tumor from a patient with metastatic disease) and 4 non-metastatic tumors as well as established paired cell lines HOS and 143B. As shown in Fig. 1c and d, sFRP2 is significantly upregulated in the primary bone tumor from patients with metastatic disease compared to tumors from patients with only localized disease, and within the highly metastatic human osteosarcoma cell line 143B compared to its paired cell line HOS that has low metastatic potential. In summary, these results provide evidence supporting increased expression of sFRP2 in metastatic OS.

Besides the enhanced expression observed in metastatic OS, using the publically available R2 database (http://hgserver1.amc.nl/cgi-bin/r2/main.cgi), we investigated whether sFRP2 expression also has clinical relevance for sarcomas, as increased mRNA expression levels are significantly associated with poorer event free and overall survival for Ewing sarcoma, the second most common pediatric bone tumor (Additional file 1: Figure S1b and c).

### sFRP2 overexpression enhances osteosarcoma cell migration and invasion in vitro

**Fig. 2:**
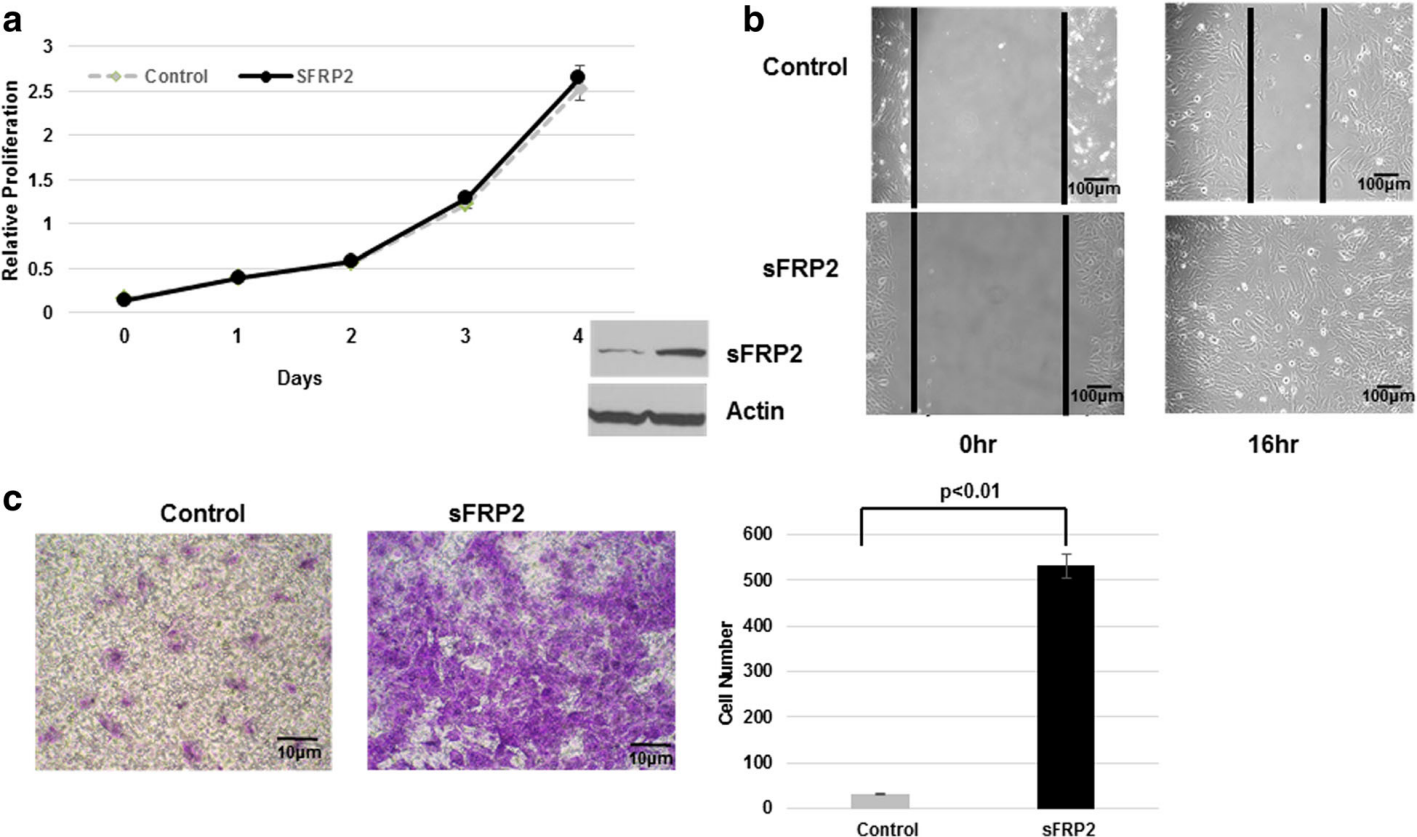
Enhanced sFRP2 expression in non-metastatic mouse OS cells does not alter proliferation, but promotes increased migratory and invasive properties. **a** CCK-8 cell proliferation assay of sFRP2/RF43 and vector control cells. Western blot analysis of whole cell lysates showing expression of sFRP2 in control and sFRP2-overexpressing cells. Actin shown as loading control. **b** Cell migration assay of sFRP2/RF43 and control RF43 cells. **c** Transwell invasion assay of sFRP2/RF43 and control RF43 cells. Crystal violet staining for control and sFRP2 expressing cells is shown. Quantification of tumor cell invasion is shown to the right

**Fig. 3:**
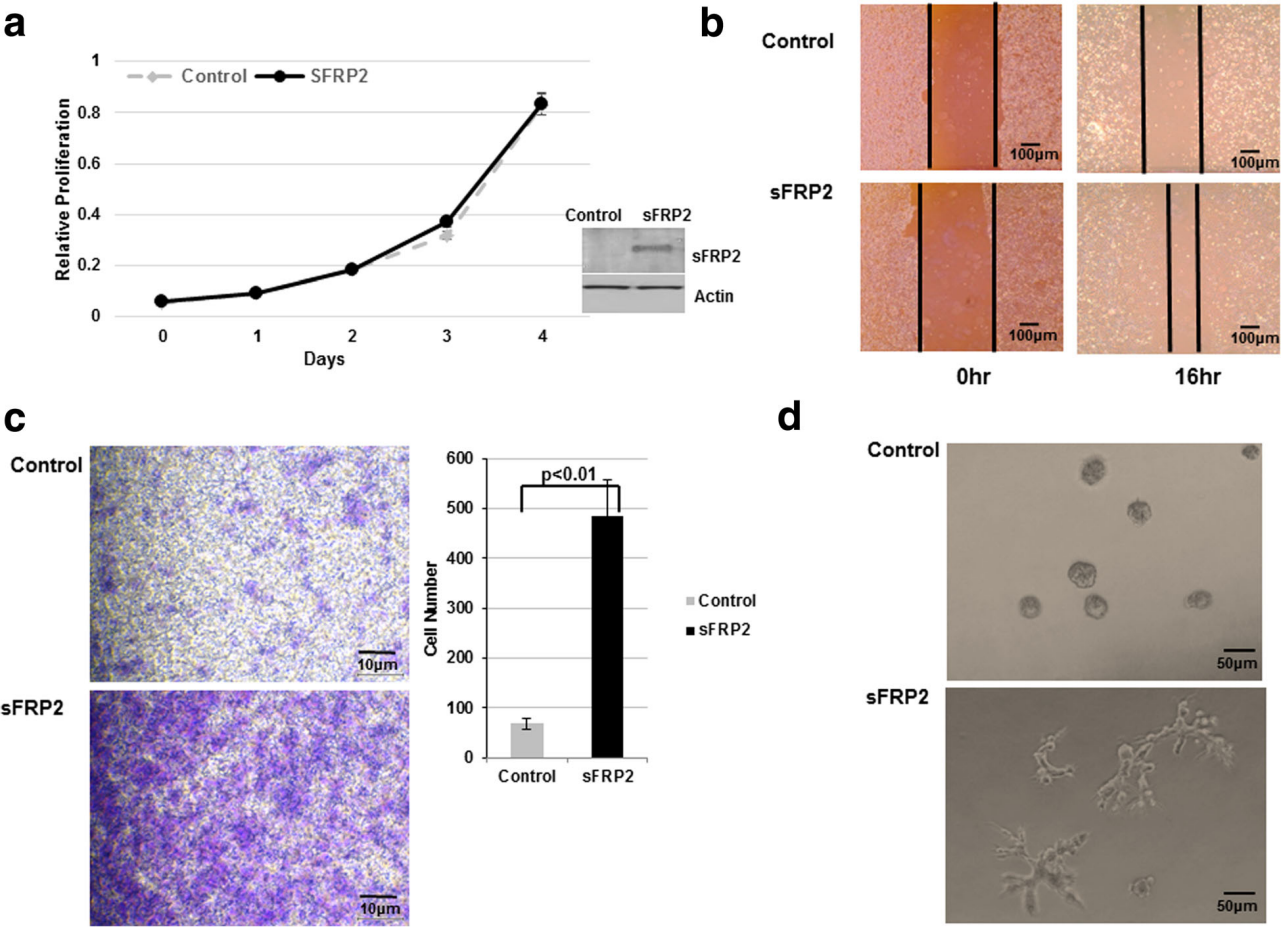
Overexpression of sFRP2 in low metastatic human OS cells drives increased invasive potential. **a** CCK-8 cell proliferation assay of sFRP2/HOS and HOS vector control cells. Western blot analysis of whole cell lysates showing expression of sFRP2 in control and stable clones. **b** Cell migration assay of sFRP2/HOS and control HOS cells. **c** Transwell invasion assay of control HOS (top) and sFRP2/HOS (bottom) cells. Crystal violet staining for control and sFRP2 expressing cells is shown. Quantification of tumor cell invasion is shown to the right. **d** 3D matrigel assay for vector control (top panel) and sFRP2/HOS cells (bottom panel)

After identifying and confirming differential expression of sFRP2 in mouse and human metastatic OS, and its clinical relevance for patients with bone sarcomas, our goal was to elucidate the phenotypic effects of sFRP2 on osteosarcoma. We expressed sFRP2 in low-metastatic mouse and human osteosarcoma cell lines, RF43 (designated as sFRP2/RF43) and HOS (designated as sFRP2/HOS). As shown in Figs. 2 and 3, cell proliferation assays for both mouse and human showed similar proliferation rates comparing control and overexpression of sFRP2 (Figs. 2a and 3a). Interestingly, sFRP2/RF43 and sFRP2/HOS revealed enhancement of invasive and migratory potential by scratch and transwell assays when compared with control cells (Figs. 2b,c and 3b,c). Furthermore, when placed in 3D matrigel culture assay, we observed sFRP2/HOS cells demonstrated enhanced invasive potential (Fig. 3d). Therefore, our data provide convincing evidence that enhanced, dysregulated expression of sFRP2 has pro-invasive properties.

### SFRP2 knockdown in metastasis osteosarcoma cell line decrease cells migration and invasion in vitro

**Fig. 4:**
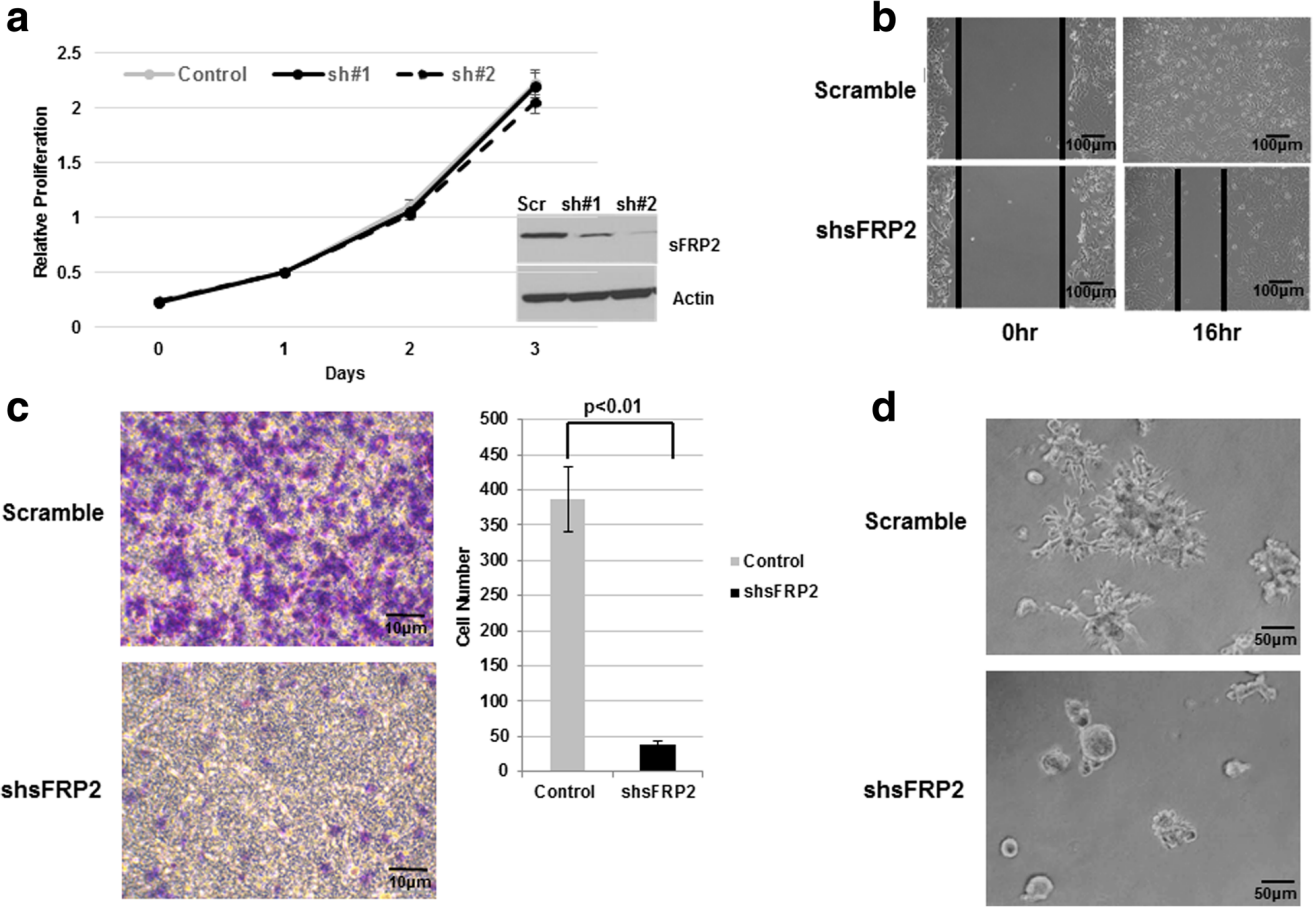
Knockdown of sFRP2 in metastatic mouse cells decreases in vitro migratory and invasive potential for OS. **a** CCK-8 cell proliferation assay of multiple shsFRP2/RF1044 and RF1044 vector control cells. Western blot analysis showing expression of sFRP2 in control and knockdown cells. **b** Cell migration assay of shsFRP2/RF1044 and control RF1044 cells. **c** Transwell invasion assay of control RF1044 (left panel) and shsFRP2/RF1044 (right panel) cells. Crystal violet staining for control and shsFRP2 expressing cells is shown. Quantification of tumor cell invasion is shown to the right. **d** 3D matrigel assay for vector control (top panel) and shsFRP2/RF1044 cells (bottom panel)

After demonstrating that enhanced sFRP2 expression contributes towards OS migratory and invasive potential in vitro, we next investigated the effects of reducing sFRP2 in highly metastatic osteosarcoma cells. After generation of stable knockdown of sFRP2 in two independent metastatic mouse osteosarcoma cell lines, RF1044 and RF379L, cell proliferation assays showed similar proliferation profiles in knockdown and control cell lines (Fig. 4a, Additional file 2: Figure S2a). However, knockdown of sFRP2 in highly metastatic mouse cells showed decreased invasive and migratory potential by scratch and transwell assays when compared with control cells (Fig. 4b,c, Additional file 2: Figure S2b,c). Furthermore, matrigel 3D culture assay of sFRP2 knockdown RF1044 cells also revealed reduced projections and decreased invasive potential compared with control cells (Fig. 4d). Therefore, our results demonstrate that decreased levels of sFRP2 decrease migratory and invasive ability of OS cells. Overall our in vitro data provide sound evidence that alterations in sFRP2 expression do not alter proliferation profiles of OS cells, but contribute towards metastatic properties through modification of migratory and invasive properties.

### SFRP2 overexpression enhanced osteosarcoma metastatic potential in vivo

**Fig. 5:**
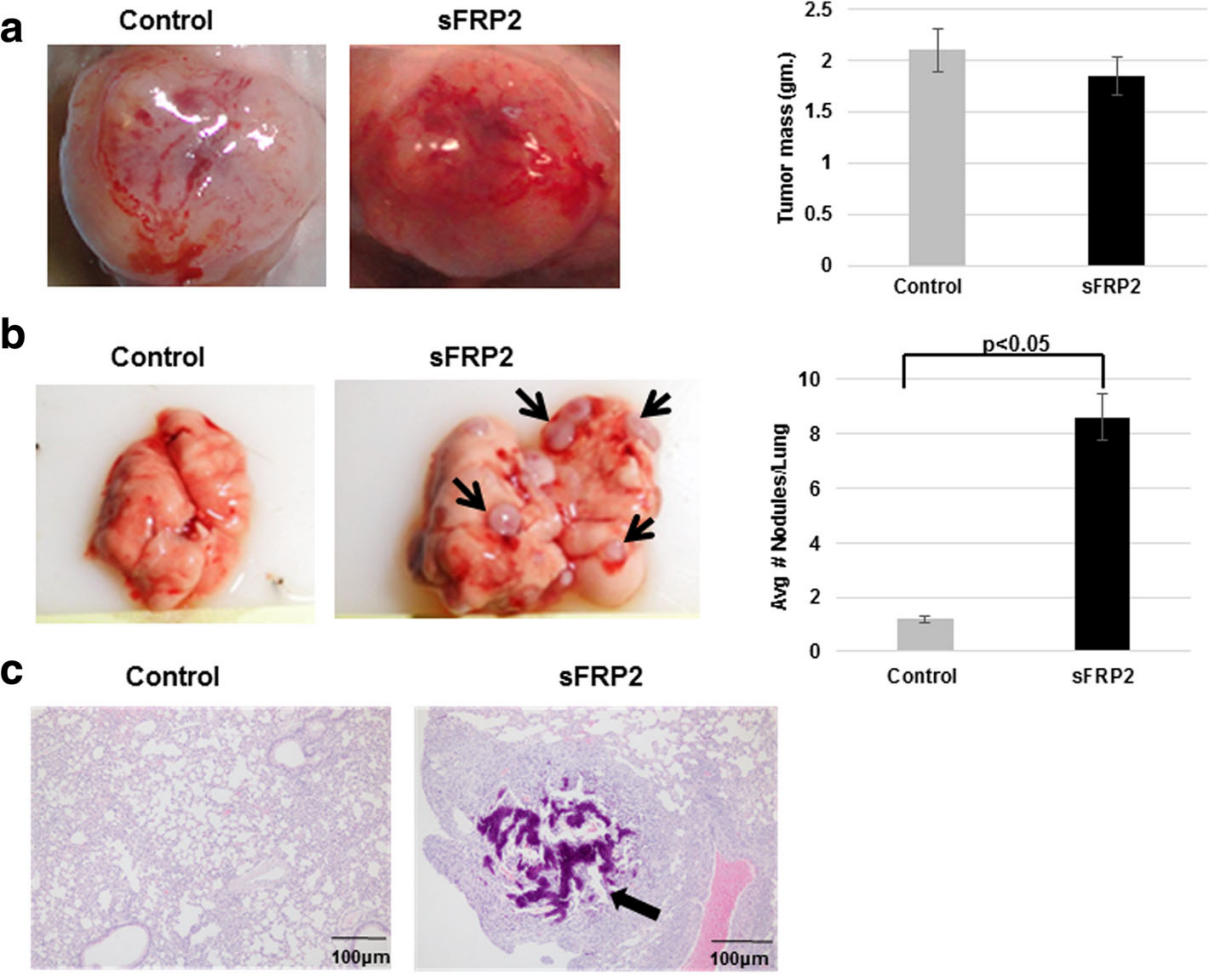
sFRP2 promotes OS metastasis, but not primary tumor growth, in vivo. **a** Representative images of primary implanted tumors and tumor mass (in grams) sacrificed at 8 weeks for control RF43 and sFRP2/RF43 cells. **b** Representative gross images of lungs from RF43 control and sFRP2/RF43 injected mice (left panels, black arrows indicate macroscopic lung lesions). Quantification of lung nodules shown in graph (right panel) (**c)**. Representative H&E images from lungs of RF43 control and sFRP2/RF43 injected mice. Black arrow indicates metastatic lung nodule

Overall our in vitro studies clearly highlight the prominent effects sFRP2 has on migratory and invasive properties. However, we next wanted to determine the effects of sFRP2 expression on OS cells in vivo. Subcutaneous injection of sFRP2/RF43 cells was performed in nude mice and tumor growth was evaluated. At 8 weeks after transplantation, average size and weight of vector control RF43 and sFRP2/RF43 tumors were not statistically different (Fig. 5a). However, comprehensive gross examination of sFRP2/RF43 mice showed increased development of distal lung metastatic lesions. Lung metastases were identified in mice in sFRP2/RF43 mice but minimal evidence of metastatic disease was found in the control group (Fig. 5b). H&E images of a lung metastasis is shown in Fig. 5c, clearly showing a prominent metastatic OS nodule.

## Discussion

The role of the secreted Frizzled related family of proteins has predominantly been associated with regulation of the Wnt signaling pathway. Specifically, studies in several malignances have demonstrated downregulation of sFRP2 via epigenetic promoter hypermethylation, suggesting a possible role as a tumor suppressor [26, 27]. However, recently reports provide evidence that sFRP2 can enhance tumor aggressiveness or progression in several cancers, including melanoma, renal cancer, angiosarcoma, and breast cancer. Specifically within angiosarcoma, studies have shown that sFRP2 enhances tumorigenesis as inhibitory antibodies directed towards sFRP2 have demonstrated anti-tumor effects [21]. In addition, sFRP2 can contribute towards therapeutic resistance through indirect activation of beta-catenin and non-canonical pathways [24].

Interestingly, sFRP2 has been reported to have a critical role in mesenchymal stem cell biology, with induced expression observed in MSCs by activation of the PI3K-Akt pathway [28, 29]. Furthermore, induction of sFRP2 enables maintenance of mitochondrial function and provides anti-apoptotic effects [29] as well as paracrine effects involved in myocardial survival and repair [30].

However the role of sFRP2 in mesenchymal tumor biology, such as osteosarcoma, remains to be elucidated. A recent report of sFRP2 suggests it provides anti-invasive functions in OS, but this report provides little direct evidence of sFRP2 function [31]. Within this report, anti-invasive properties were only demonstrated after use of 5’aza-cytidine, or decitabine, a broad, non-specific hypomethylating agent that can cause significant global alterations in cellular gene regulation.

We believe our in vitro and in vivo studies provide direct molecular evidence that sFRP2 expression significantly contributes to OS metastatic phenotype. In our studies, we have demonstrated that sFRP2 has enhanced expression in metastatic mouse and human osteosarcomas. We specifically focused on the direct biological effects of sFRP2 on OS by using gain and loss-of-function experiments that revealed sFRP2 expression has no observable effect on tumor cell proliferation both in vitro and in vivo. In contrast, stable expression of sFRP2 significantly promotes migratory and invasive potential of OS cells both in vitro *and* in vivo. While we report that gain-of-function sFRP2 enhanced metastatic potential in vivo, when we attempted to silence sFRP2 expression in metastatic OS cells in xenograft models, we obtained only modest decrease of metastatic potential (data not shown). This suggests that enhanced sFRP2 expression may be sufficient to drive metastatic phenotype, but other factors, including alterations in other Wnt modulators (as reported in our prior publication) [15] may be compensating for the knockdown of sFRP2 in vivo. Further molecular studies are warranted, including a more analysis of the in vivo sFRP2 knockdown tumors (versus control) might provide insights into compensatory mechanisms ongoing in vivo that could still be driving the metastatic state.

While we believe our studies provide significant contributions towards understanding metastatic mechanisms for osteosarcoma, further studies are warranted to investigate direct downstream molecular mechanisms associated with sFRP2 expression. In order to investigate a possible mechanism of the sFRP2-dependent phenotype, we performed Wnt signaling reporter studies upon alteration of sFRP2 in the OS cells, but observed no statistically significant changes in canonical Wnt activation (data not shown). Investigations into additional signaling cascades could provide novel insights into the direct role of sFRP2 and metastatic mechanisms for osteosarcoma. Besides the aforementioned PI3K-Akt pathway, additional candidate mechanisms include the possibility that sFRP2 could positively regulate the expression of the stem cell transcription factor Slug [32], which is involved promoting the metastatic phenotypes of carcinomas [33]. Studies into if sFRP2 could alter the differentiation or stemness of osteosarcoma cells would also be interesting to investigate.

Finally, using our pre-clinical engineered and orthotopic mouse models, investigations into the ability to therapeutically target sFRP2 within OS would be extremely interesting. Presently there are pre-clinical monoclonal antibodies targeting sFRP2 [21], which could provide biological evidence for the role of this molecule on sarcoma progression.

## Conclusions

While many of the negative regulators of Wnt signaling pathway have been implicated in inhibiting the development and progression of OS, we have identified and characterized a sFRP family member, specifically sFRP2, which is enhanced within metastatic OS. Our studies provide significant insights into a potential unique role for sFRP2 as a novel pure driver of invasive and metastatic phenotypes for this disease. Both our in vitro and in vivo gain and loss-of-function studies indicate that sFRP2 expression has no significant effects on tumor cell proliferation, but clearly dictates invasive and migratory phenotypes and enhances metastatic potential. We believe these studies will provide the foundation for additional molecular studies as well as avenues for novel therapeutic intervention.

## Additional files

Additional file 1: Figure S1. Relative sFRP2 expression across panel of tumors and sarcoma survival outcomes associated with sFRP2 expression. a. TCGA data comparing relative expression of sFRP2 (cBioPortal for Cancer Genomics, http://www.cbioportal.org) across a panel of different tumor types demonstrating high expression in sarcomas (8th of 26 tumor types). b and c. EFS and overall survival scans for sFRP2 derived from clinically annotated human Ewing sarcoma gene expression database (http://hgserver1.amc.nl/cgi-bin/r2/main.cgi). High expression (blue line) and low expression (red line). (PDF 330 kb)
Additional file 2: Figure S2. Knockdown of sFRP2 in metastatic mouse cells decreases in vitro migratory and invasive potential for OS a. CCK-8 cell proliferation assay of shsFRP2/RF379L and RF379L vector control cells. Western blot analysis of whole cell lysates showing expression of sFRP2 in control and knockdown cells. b. Cell migration assay of shsFRP2/RF379L and control RF379L cells. c. Transwell invasion assay of control RF379L (top panel) and shsFRP2/RF379L (bottom panel) cells. (PDF 203 kb)

## Abbreviations

CCK-8: Cell-counting kit-8
DMEM: Dulbecco’s modified eagle’s medium
EFS: Event-free survival
FBS: Fetal bovine serum
GEMM: Genetically engineered mouse model
LEF: Lymphoid enhancer factor
MSC: Mesenchymal stem cells
Nkd2: Naked2
OS: Osteosarcoma
PBS: Phosphate-buffered saline
qPCR: Quantitative polymerase chain reaction
sFRP2: Secreted frizzled-related protein 2
TCF: T cell factor
Wnt: Wingless signaling pathway
