# Supplementary figures and images for: Secreted Frizzled-Related Protein 2 (sFRP2) promotes osteosarcoma invasion and metastatic potential
Source: BMC Cancer. 2016 Nov 8;16:869. doi: 10.1186/s12885-016-2909-6 (PMC5100268; doi:10.1186/s12885-016-2909-6)

# Supplemental Figure 1

**a**

SFRP2 expression -- RNA Seq V2 (log2)

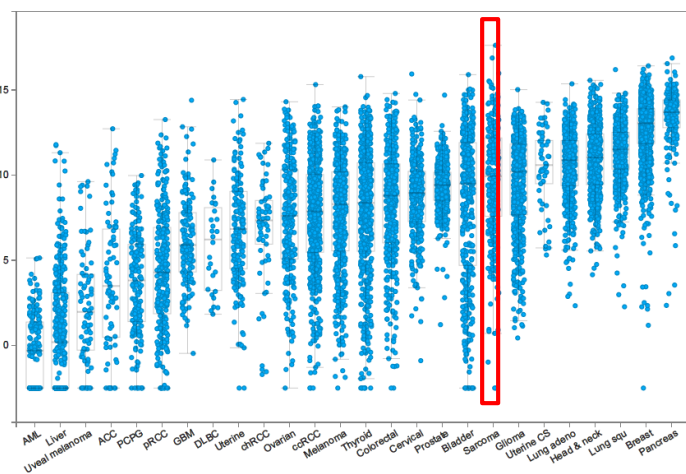

**b**

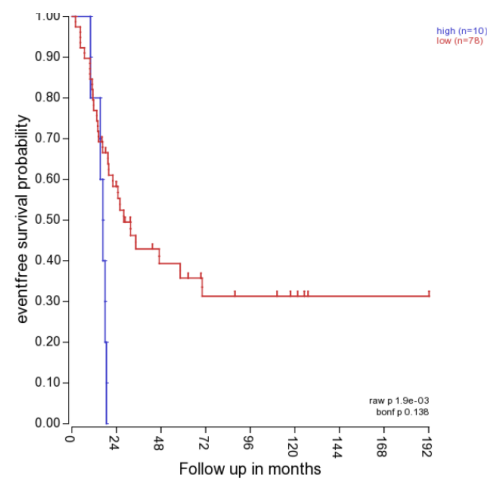

**c**

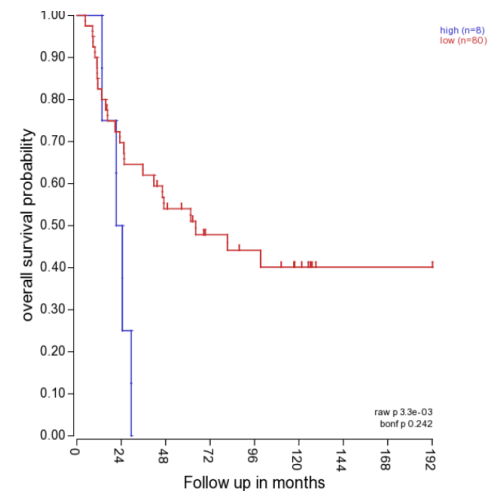

Supplement: Additional file 1: Figure S1. — Relative sFRP2 expression across panel of tumors and sarcoma survival outcomes associated with sFRP2 expression. a. TCGA data comparing relative expression of sFRP2 (cBioPortal for Cancer Genomics, http://www.cbioportal.org) across a panel of different tumor types demonstrating high expression in sarcomas (8th of 26 tumor types). b and c. EFS and overall survival scans for sFRP2 derived from clinically annotated human Ewing sarcoma gene expression database (http://hgserver1.amc.nl/cgi-bin/r2/main.cgi). High expression (blue line) and low expression (red line). (PDF 330 kb) [file 12885_2016_2909_MOESM1_ESM.pdf]

## Supplemental Figure 2

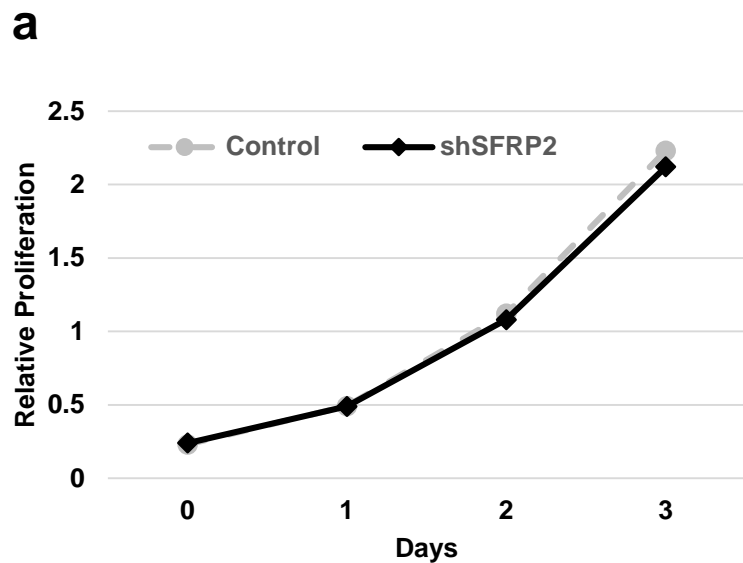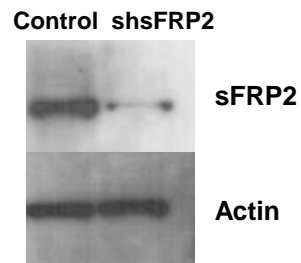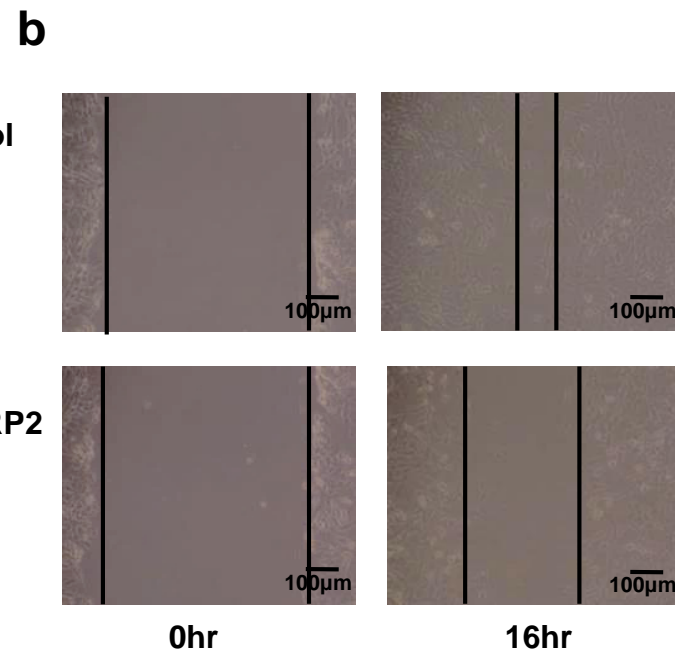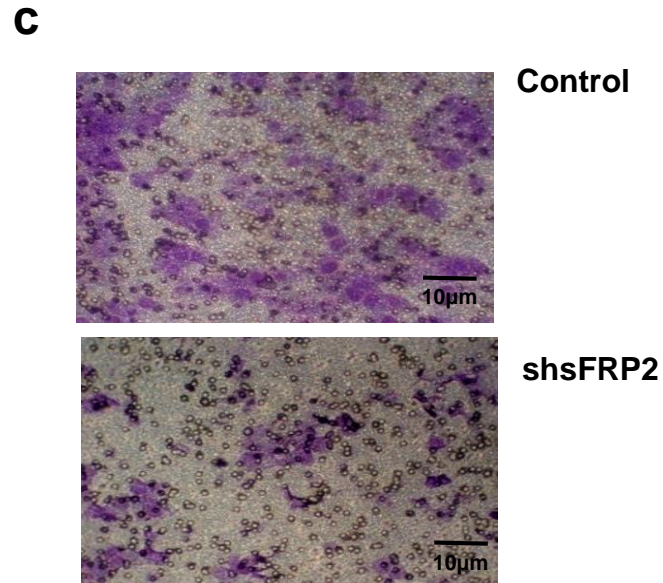

Supplement: Additional file 2: Figure S2. — Knockdown of sFRP2 in metastatic mouse cells decreases in vitro migratory and invasive potential for OS a. CCK-8 cell proliferation assay of shsFRP2/RF379L and RF379L vector control cells. Western blot analysis of whole cell lysates showing expression of sFRP2 in control and knockdown cells. b. Cell migration assay of shsFRP2/RF379L and control RF379L cells. c. Transwell invasion assay of control RF379L (top panel) and shsFRP2/RF379L (bottom panel) cells. (PDF 203 kb) [file 12885_2016_2909_MOESM2_ESM.pdf]
